# Navigating the Colorectal Cancer Maze: Unveiling Pathways To Diagnosis, Management, Pathophysiology and Prevention

**DOI:** 10.1007/s11912-025-01707-w

**Published:** 2025-08-09

**Authors:** Khalid Ali Mohammed Al Kamzari, Constantina Constantinou

**Affiliations:** Department of Basic and Clinical Sciences, University of Nicosia Medical School, 21 Ilia Papakyriakou, P.O. Box 24005, Engomi, Nicosia, 2414, CY-1700 Cyprus

**Keywords:** Colorectal cancer, Epidemiology, Pathophysiology, Management, Vaccine-based immunotherapy

## Abstract

**Purpose of Review:**

Colorectal cancer is the third most prevalent cancer globally and the second leading cause of cancer-related mortality. It typically develops over years through the progression of benign polyps to malignancy, driven by genetic alterations—either spontaneous or inherited. This review summarizes current knowledge on colorectal cancer, including its epidemiology, risk factors, diagnostic methods, treatment strategies, preventative measures, and research developments, while identifying knowledge gaps to guide future studies.

**Recent Findings:**

Colorectal cancer is influenced by numerous lifestyle-related risk factors, such as high-calorie diets, processed foods, red meat, smoking, obesity, and alcohol use. Colonoscopy, imaging tests, and biopsies remain essential for diagnosis, while the TNM staging system continues to guide therapeutic decisions. Treatment options range from early-stage surgical interventions to chemotherapy, radiotherapy, and targeted therapies in advanced stages, with neoadjuvant and adjuvant treatments offering improved outcomes. Experimental therapies, including regorafenib and cancer vaccines, are under investigation. Prevention strategies focus on healthy lifestyles and risk avoidance, alongside screening techniques including fecal occult blood tests, colonoscopy, and sigmoidoscopy. Screening programs emphasize individuals with genetic susceptibility, and clinical trials aim to enhance both screening and therapeutic approaches.

**Summary:**

Colorectal cancer poses a substantial global health challenge. Advances in diagnostics, treatment, and prevention are promising, but further research is needed to improve management strategies and address gaps in screening. Lifestyle changes and early detection through targeted screening remain critical for reducing the disease burden worldwide.

## Introduction

Colorectal cancer (CRC) is the third most commonly diagnosed cancer and ranks as the second leading cause of cancer-related deaths among both women and men in the US [1]. The risk factors associated with CRC include non-modifiable risk factors such as age, with individuals aged 50 and older facing increased risk, as well as genetic predispositions. Additionally, modifiable risk factors contribute significantly to the development of CRC and include unhealthy diets, smoking, physical inactivity, excessive alcohol consumption and obesity [2]. Recent evidence indicates that an imbalance in gut microbiota could also play a role in the carcinogenesis of CRC [3, 4].

CRC develops through the sequential transformation of healthy mucosa into an invasive tumor. This stepwise progression underscores the significance of timely interventions, particularly through screening programs, which facilitate the identification of tumors in their early stages when they are more likely to be treatable [5]. However, most cases are discovered at advanced stages, leading to delayed treatments and resulting in poor prognosis [6].

Diagnosis of CRC involves a combination of physical examinations, laboratory tests, and imaging techniques. Ultrasound and CT scans are used to detect abdominal masses. Endoscopic methods, such as colonoscopy and capsule endoscopy, allow for the direct visualization of the colon and are instrumental in detecting polyps or tumours facilitate biopsy collection. Colonoscopy facilitates biopsy collection for histopathological examination, which is essential for confirming a cancer diagnosis. Capsule endoscopy, while effective in visualizing the gastrointestinal tract, does not allow for tissue biopsy. Together, these diagnostic tools help assess the presence of a tumour, determine its stage, and guide treatment decisions [7].

In early-stage CRC, surgery is the primary treatment, aiming to remove the lesion and prevent metastasis. In advanced stages, chemotherapy and radiotherapy are often employed to shrink tumours, enhancing their resectability and potentially improving surgical outcomes. When surgery is not an option, these treatments play a crucial role in palliation, slowing disease progression, and improving quality of life [7, 8].

**Fig. 1:**
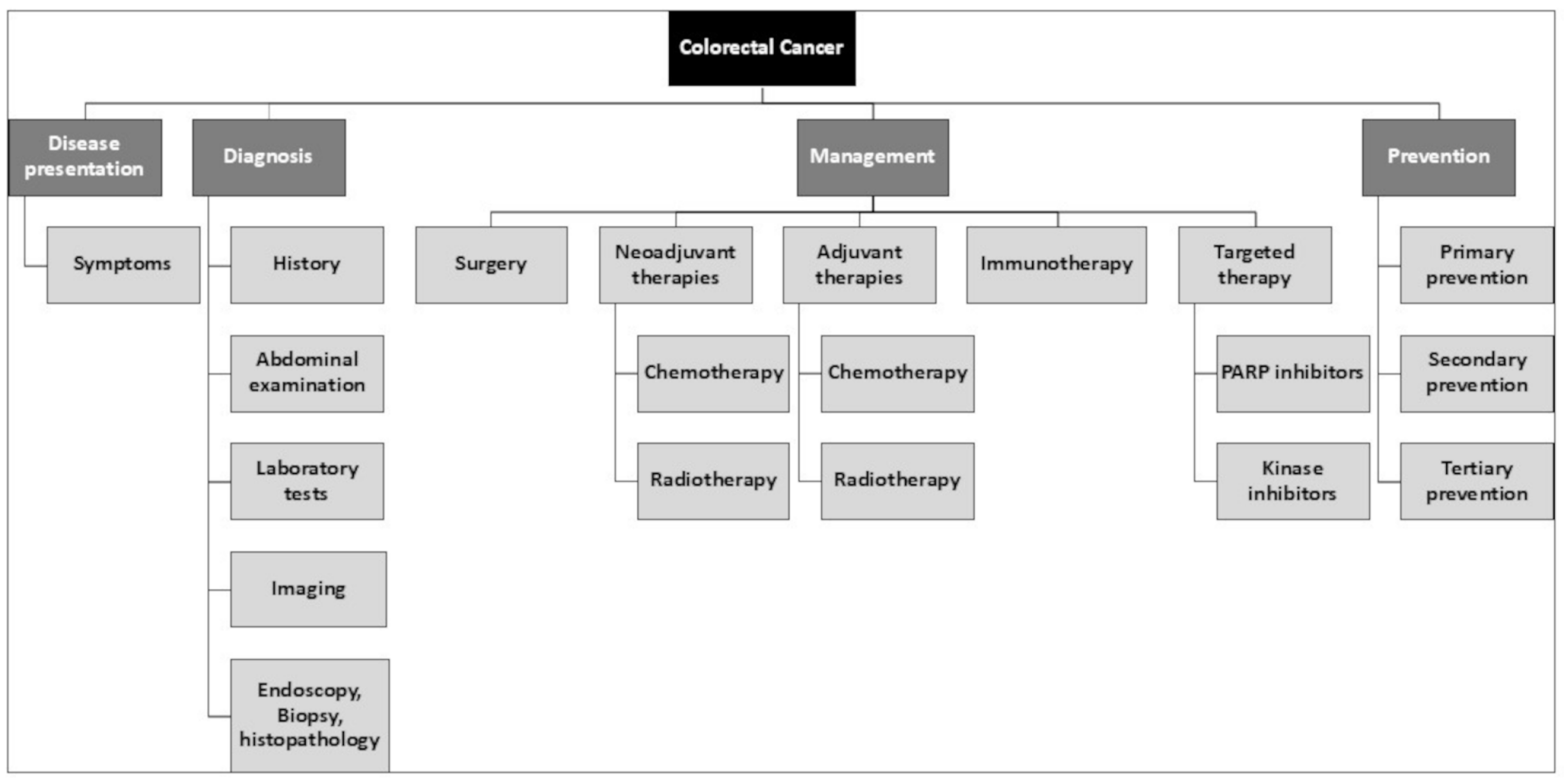
Comprehensive overview of colorectal cancer including clinical presentation, diagnostic process, management and preventive strategies

This narrative literature review aims to provide a comprehensive overview of recent research findings on CRC. It systematically examines key aspects of the disease, including its presentation, epidemiology, diagnosis, pathophysiology, management, and prevention. As illustrated in Fig. 1, the review encompasses these interconnected domains, offering a framework for understanding the multi-dimensional nature of CRC. By analyzing these areas, the review seeks to enhance our knowledge of the disease, contributing to evidence-based approaches in both research and clinical practice.

## Methods

This narrative review primarily utilized scientific literature from databases such as PubMed, Medline Complete, and Cochrane. The search incorporated keywords including ‘colorectal cancer,’ ‘epidemiology,’ ‘diagnosis,’ ‘management,’ ‘adjuvant therapies,’ ‘neo-adjuvant therapies,’ ‘tumorigenesis,’ and ‘prevention strategies,’ using Boolean operators (e.g., “Colorectal cancer” AND “Diagnosis”). Most selected studies were published between 2014 and 2024, with some exceptions. Only English-language publications were included. Additional data were sourced from reputable organizations, including the WHO, GLOBOCAN, the National Cancer Centre, and Bowel Cancer UK. Guidelines from the National Institutes for Health and Care Excellence (NICE, UK) were also reviewed, and clinical trial data were retrieved from ClinicalTrials.gov.

### Disease Presentation

In early-stage CRC, the disease is usually asymptomatic, but as it progresses, faecal blood may be visible, varying in color based on the bleeding site. This can lead to iron deficiency anemia, with symptoms such as weakness, fatigue, and paleness [9]. Advanced-stage symptoms include intestinal obstruction, causing abdominal cramps, distension, and vomiting, with large masses sometimes being palpable [9]. Bowel movement changes, such as constipation, diarrhea, and stool narrowing, may also occur. In late stages, systemic symptoms including weight loss and fatigue are common [2]. While these symptoms suggest CRC, their specificity varies. Rectal bleeding has a positive predictive value (PPV) of 4.0% and a specificity of 99.4%, yet lower abdominal pain and bloating are less specific and have a lower PPV [10].

### Diagnosis

#### History

Gathering a complete patient history is critical for identifying predisposing factors and symptoms that may indicate a high probability of CRC and require further investigations to confirm the diagnosis [11].

#### Abdominal Physical Examination

The next step in diagnosing CRC involves an abdominal physical examination, which can indicate the presence of a mass or obstruction in the gastrointestinal (GI) tract. In females, the vagino-recto-abdominal examination is additionally used to investigate for any vaginal metastasis. Additionally, a rectal examination is also considered to assess any masses in the rectum [12].

#### Laboratory Tests

Laboratory tests play a pivotal role in the diagnosis of CRC. A complete blood count is performed to detect anaemia, which can be indicative of chronic GI bleeding, and stool testing for occult blood is undertaken as it may indicate lesions in the GI tract. Additionally, liver and renal function tests are assessed to evaluate organ functionality [13].

In cases where CRC is suspected, tumour biomarkers are measured to aid in diagnosis and disease monitoring. Carcinoembryonic antigen (CEA) is the most widely utilized biomarker for CRC, particularly for monitoring disease progression and response to treatment. Alpha-fetoprotein (AFP), while primarily associated with liver cancers, may occasionally be measured to evaluate for potential liver metastases. Additionally, CA125 is used in cases where ovarian or peritoneal metastases are suspected [13].

#### Imaging

Initial imaging techniques are crucial for the detection and evaluation of CRC. CT imaging is the preferred modality for assessing intra-abdominal metastases due to its superior sensitivity and detailed visualization of abdominal organs and lymph nodes. For rectal cancer, rectal MRI is the preferred imaging technique where available, as it provides superior anatomical detail and accurately assesses tumour depth and potential involvement of surrounding structures. Furthermore, barium gas double X-ray is employed to identify filling defects and mucosal degradation in the intestine [12].

#### Endoscopy, Biopsy and Histopathology

Following laboratory tests and initial imaging, endoscopic procedures are conducted to evaluate CRC in detail. These procedures included sigmoidoscopy, rectoscopy, and colonoscopy, which help assess tumor size, depth, location, infiltration, and morphology. During endoscopy, biopsies of any suspicious lesions are taken for histopathological examination, which is essential for confirming the diagnosis and guiding treatment plans [12, 14].

Although colonoscopy is an invasive procedure, less invasive alternatives, such as capsule endoscopy, have been developed to examine nearly the entire GI tract. This technique offers a more patient friendly option while still providing valuable diagnostic information [8, 15].

#### Genetic Testing

Several mutations in genes including *APC*, *NRAS*, *KRAS*, *TP53* and *BRAF* contribute to CRC pathogenesis. While testing for these genetic mutations provides valuable information for guiding treatment strategies and assessing prognosis, it is not diagnostic of CRC. Testing for mismatch repair (MMR) protein expression or microsatellite instability (MSI) is often conducted to identify specific tumour characteristics and guide therapeutic decisions, such as the use of immunotherapy. Although research into chromosomal instability (CIN) and the use of microRNAs as prognostic or predictive markers is ongoing, these tools are not yet widely used in clinical practice [16].

#### Staging

The staging of CRC occurs in two phases. and follows the TNM classification system, which assesses tumour depth (T), lymph node involvement (N), and distant metastases (M). Patients are grouped into four stages (I-IV), each corresponding to a specific prognosis. Clinical staging is conducted after diagnosis and prior to initiating treatment. It uses imaging studies (e.g., CT, MRI, PET-CT) and, where needed, endoscopy to estimate the extent of the disease. This phase is critical for guiding pre-treatment decisions, such as the use of neoadjuvant therapy. Pathological staging is performed after surgical resection to provide a definitive assessment of the primary tumour, lymph nodes, and metastatic involvement through histopathological evaluation [7].

For patients undergoing neoadjuvant therapy, post-treatment staging assesses tumour response and refines subsequent treatment decisions. While less frequently used, the Modified Astler-Coller and Dukes systems may also be referenced. Ultimately, treatment decisions consider not only the cancer stage but also patient comorbidities and preferences [7].

### Diagnosis of Complex Gastrointestinal Cases

In complex GI cases where diagnosis is challenging, investigative approaches may include the use of open surgery and laparoscopic exploration. Additionally, surgical intervention may be necessary in instances of suspected bowel perforation, intestinal obstruction or significant GI hemorrhage [6, 17].

### Emerging Technologies

Emerging technologies are increasingly incorporating artificial intelligence (AI) into different areas of healthcare. AI can gather and analyze public data for digital epidemiology, assisting clinicians in early disease detection and health surveillance. Furthermore, AI can enhance the diagnostic process of CRC by improving imaging techniques, allowing faster image interpretation, and minimizing artefacts. Moreover, AI can play a crucial role in the detection and classification of CRC based on histopathology [18].

### Epidemiology

CRC was the third most common cancer globally in 2020, impacting individuals of all genders and ages [19].

#### Incidence

In 2022, the global age-standardized incidence rate (ASIR) for CRC was 19.7 per 100,000 individuals. Marked regional differences were observed, with Europe and Oceania reporting the highest ASIRs, ranging between 30 and 36 per 100,000, followed by North America, where the rate was approximately 26–30 per 100,000. Latin America, the Caribbean and Asia had intermediate rates, estimated between 12 and 20 per 100,000. The lowest incidence was recorded in Africa, with an ASIR of approximately 5 to 9 per 100,000. Notably, the ASIR was higher in males (22.0 per 100,000) compared to females (15.2 per 100,000) [19].

#### Prevalence

In 2022, the global 5-year prevalence of CRC was approximately 73.2 per 100,000 individuals. Regional disparities were evident, with Central Europe exhibiting prevalence of 90–100 per 100,000 and North America 80–95 per 100,000 individuals [19].

#### Survival

According to global cancer survival estimates reported by the CONCORD-3 study and other international sources, the overall global 5-year survival rate for CRC varies widely by region but is estimated to be 60% for all stages. Survival rates differ significantly depending on the stage at diagnosis, emphasizing the crucial role of early detection. The global 5-year relative survival rate for localized (stage I) CRC is approximately 90%, while for regional (stage II and III) CRC, it drops to about 70–75%. For distant (stage IV) CRC, the 5-year relative survival rate decreases dramatically to around 15% [20].

#### Mortality

CRC ranked the third leading cause of cancer-related deaths worldwide in 2022, accounting for approximately 1,050,000 deaths globally [19]. The global age-standardized mortality rate (ASMR) for CRC was around 10.0 deaths per 100,000 population. Colon and rectal cancers contributed approximately 6.0 and 4.0 deaths per 100,000, respectively. Regionally, the highest mortality rates were observed in Asia, with an ASMR of about 11.5 deaths per 100,000, representing roughly 55% of global CRC deaths. Europe followed with an ASMR near 14.0 deaths per 100,000, while Latin America and the Caribbean reported rates around 7.0 deaths per 100,000 [19].

CRC is expected to escalate, with an estimated 3.2 million new cases and 1.6 million deaths by 2040. This projection indicates that the global impact of CRC will significantly increase. Notably, highly developed countries are anticipated to have most of these cases [21].

### Pathophysiology

#### Anatomical Location of CRC

CRC can develop anywhere along the colon and rectum, from the cecum to the rectum. The distribution of CRC varies by anatomical site. A study involving 3,058 cases of CRC found the following distribution: sigmoid colon 24.5%, ascending colon 20.7%, rectum 17.3%, cecum 15.5%, transverse colon 8.2%, descending colon 7.2%, and rectosigmoid junction 6.6%. Additionally, 7.1% lacked anatomical location information and were censored at the time of diagnosis [22].

#### Histological Types of CRC

Pathological histological types of adenocarcinomas include papillary adenocarcinoma, mucinous adenocarcinoma, adenosquamous carcinoma, tubular adenocarcinoma, signet-ring cell carcinoma, undifferentiated carcinoma, carcinoid carcinoma, and squamous cell carcinoma [9, 23–25].

#### Development of CRC

The development of CRC typically begins with hyperplasia, characterized by an increased proliferation of normal cells. Over time, hyperplasia can progress to dysplasia, which involves abnormal cellular architecture and atypical growth, marking a precancerous stage. Dysplastic changes may further advance to the formation of adenomas, some of which can eventually transform into invasive cancer. Chromosomal instability, genetic mutations, and hypermethylation of CpG islands in gene promoter regions are three well-established molecular mechanisms associated with CRC pathogenesis [16].

### Theories for the Development of CRC

Several theories have emerged to explain the development of CRC.

#### The Adenoma-Carcinoma Sequence

The adenoma-carcinoma sequence is a well-established theory, describing the progression of morphological changes from hyperplasia to dysplasia leading to the formation of malignant, invasive tumours. This process is primarily characterised by failure in the regulation of cell proliferation [16, 26, 27]. The conventional pathway begins with mutations in the adenomatous polyposis coli (*APC*) gene, which promotes increased cell proliferation and the development of a polyp. This is followed by sequential mutations in the genes *KRAS* and *DCC*, leading to further proliferation and increasing the size of the polyp. Furthermore, the mutation in the *TP53* tumour suppressor gene contributes to carcinogenesis, and the malignant tumour formed spreads to the tissue surrounding it and to the distant organs [16, 28, 29].

#### The Mutator Pathway

The mutator pathway suggests that certain genetic syndromes which strongly elevate the risk from CRC, result from inherited genetic mutations [16, 30]. These syndromes include the Familial Adenomatous Polyposis (FAP) syndrome (which is associated with a mutation in the *APC* gene), the Lynch syndrome (LS), the Peutz-Jeghers syndrome, hereditary mixed polyposis, juvenile polyposis, and MUTYH-associated polyposis [16, 31, 32].

#### The Serrated Pathway

The serrated pathway involves hyperplastic polyps and a subgroup of serrated polyps as precursors to CRC [16, 30]. It is characterized by cytosine hypermethylation in CIMP lesions, *BRAF* mutations, promoter methylation, and microsatellite instability (MSI) [16, 35]. Sessile serrated adenomas/polyps linked to this pathway are significant CRC precursors. Serrated polyposis syndrome, characterized by numerous large, serrated polyps, is also a risk factor for CRC [16, 33, 34].

### Genetic and Epigenetic Mutations Associated with CRC

CRC is a cancer characterized by genetic and epigenetic mutations. Mutations in the *KRAS* oncogene is among the most significant somatic mutations, found in approximately 50% of CRC cases [9, 35]. Allelic losses of tumor suppressor genes are common in CRC, including losses of chromosome 5q in 20%-50% of cases, chromosome 17p in over 75%, and chromosome 18q in more than 70% of CRCs [9, 35]. Mutations in the *TP53* gene may provide a selective growth advantage, promoting tumor progression. Early in colorectal tumorigenesis, DNA methylation loss contributes to genomic instability. CRC is also associated with increased C-MYC expression, elevated tyrosine kinase activity, and altered glycoconjugate expression [9, 35].

### Local Invasion and Metastasis

Four primary pathways facilitate the spread of CRC including local invasion, lymphatic metastasis, hematogenous metastasis, and implantation/metastasis. Local invasion entails tumour infiltration into surrounding tissues, while lymphatic metastasis involves the spread of cancer cells to regional lymph nodes via the lymphatic system. Hematogenous metastasis occurs when cancer cells travel through blood vessels, often targeting the liver and lungs. Implantation/metastasis involves detached cancer cells implanting in the abdomen and pelvic peritoneum, forming metastatic foci [9].

### Management

A key determinant of management strategy is staging, which is used to apply the appropriate therapy protocol that significantly varies based on the stage of the disease at the time of diagnosis [7]. The evolving landscape of CRC treatment underscores the importance of tailoring interventions based on tumor biology, offering a promising avenue for improving outcomes in the complex journey of combating CRC [8].

### Surgery

Surgery is fundamental in managing stage I-III colon and rectal cancer, requiring meticulous dissection to remove tumours and their primary lymphatic zones. Pre-operative considerations include patient age, tumour stage and tumour location [36].

### Colon Cancer Surgery

In colon cancer management, laparoscopic surgery is a safe alternative to open surgery, with laparoscopic resection recommended when appropriate. The decision depends on tumour characteristics, surgical risks/benefits, and the surgeon’s experience [8, 36].

Ostomies—such as loop ileostomy, loop colostomy, or permanent colostomy—are created to manage bowel obstruction, protect healing tissues after surgery, or prevent complications such as anastomotic leakage, especially after preoperative radiation for rectal cancer. Temporary ostomies provide faecal diversion to support healing, while permanent colostomies are used when bowel restoration is not feasible. The choice of ostomy depends on the tumour location, disease extent, and patient needs [8, 36].

### Rectal Cancer Surgery

For rectal cancer, treatment strategies vary by stage. Early-stage rectal cancer can utilize minimally invasive techniques, such as trans-anal endoscopic microsurgery, to avoid abdominal scars and stomas. Advanced tumours may require total mesorectal excision, especially after neoadjuvant therapy [8]. According to NICE guidelines, surgical treatment is tailored to the disease stage, with options including transanal excision and other minimally invasive techniques. While laparoscopic surgery is preferred, open surgery may be necessary for more complex cases. Robotic and transanal total mesorectal excision is advised within specialized settings, and extensive surgeries including multi-visceral resections may be required for advanced cases [36].

### Adjuvant and neo-adjuvant Therapies

#### Neoadjuvant Chemotherapy (NACT)

NACT involves the administration of anti-cancer drugs before the primary treatment, usually surgery to shrink the tumour and facilitate its removal [37]. Common NACT regimens include FOLFOX (folinic acid, fluorouracil, oxaliplatin) and CAPOX (capecitabine, oxaliplatin), which are primarily used for tumours without high microsatellite instability (MSI-H) or mismatch repair deficiency (dMMR) [37].

For MSI-H/dMMR tumours, immune checkpoint inhibitors such as dostarlimab and pembrolizumab (both PD-1 inhibitors) are recommended based on their efficacy in these biomarker-defined subgroups. FOLFIRI (folinic acid, fluorouracil, irinotecan) and TAS-102 (trifluridine and tipiracil), while valuable in metastatic or refractory settings, are not standard NACT regimens in the non-metastatic context [37].

NACT offers the advantage of targeting occult micrometastatic disease early, improving surgical outcomes by reducing tumour size and enhancing resection success. Treatment regimens are tailored to individual patient and tumour characteristics, including biomarker status, tumour location, and disease stage [37].

#### Neoadjuvant Radiotherapy (NART)

NART is crucial in rectal cancer treatment, especially for stage II-III cases, by shrinking tumours and improving surgical outcomes with clearer margins and reduced recurrence risk. Its use in colon cancer is rare and typically considered only for large tumours or those involving adjacent organs. Recently, combining NART with NACT, termed total neoadjuvant therapy (TNT), has become standard for locally advanced rectal cancer [37, 38].

#### Adjuvant Chemotherapy

Adjuvant chemotherapy is administered post-surgery to eliminate remaining cancer cells and reduce recurrence risk [39]. For CRC, particularly in stage II-III, common regimens include CAPOX and FOLFOX, with the choice influenced by tumour histopathology, patient condition, and preferences (NICE, 2020). These regimens, focusing on microscopic disease, are critical for improving patient outcomes [7, 36, 40].

#### Adjuvant Radiotherapy

Adjuvant radiotherapy, used primarily in rectal cancer, targets residual cancer cells post-surgery to prevent recurrence [39]. In high-risk colon cancer, it may be considered if there is a significant local recurrence risk. It aims to target any remaining microscopic disease [8].

### Immunotherapy

Immunotherapy focuses on using the immune system to fight cancer. Immune checkpoint inhibitors (ICIs), modulate interactions among T cells, antigen-presenting cells, and tumor cells. ICIs have shown significant efficacy in treating patients with metastatic CRC (mCRC) exhibiting deficient mismatch repair (dMMR) or high microsatellite instability (MSI). The FDA has approved nivolumab and pembrolizumab, alone or with ipilimumab, for such cases based on their demonstrated efficacy in clinical trials. These immune checkpoint inhibitors have shown significant and durable response rates in patients with advanced or metastatic CRC. For instance, pembrolizumab achieved an overall response rate (ORR) of 40%, with some patients experiencing sustained responses lasting over two years in clinical studies. Similarly, nivolumab, when used alone or with ipilimumab, has demonstrated durable disease control and progression-free survival benefits in this biomarker-defined subgroup [41]. The challenge remains to extend these benefits to the majority of mCRC patients with MMR-proficient and low MSI tumours, as these often lack sufficient mutated antigens for immunotherapy to target effectively [42].

### Targeted Therapy

Targeted therapy for CRC employs drugs or substances that precisely attack cancer cells, usually by interfering with specific molecules involved in tumour growth and progression. Unlike traditional chemotherapy, which can affect both cancerous and healthy cells, targeted therapy aims to minimize damage to normal cells by focusing on specific cellular targets associated with cancer [39].

#### PARP Inhibitors

Poly ADP-ribose polymerase (PARP) is an enzyme essential for repairing single-strand breaks in DNA. PARP inhibitors (PARPi) have shown marked efficacy in cancers with *BRCA1/2* mutations, such as ovarian and breast cancers, and are FDA-approved for these indications. However, their role in CRC is limited due to the low prevalence of *BRCA1/2* mutations in these tumours. Current research is investigating their use in CRC with other DNA repair deficiencies, such as homologous recombination deficiency (HRD), and in combination with other therapies, though clinical evidence in this context remains nascent [43, 44].

#### Kinase Inhibitors

Kinases are enzymes essential for cell signalling, growth, and survival with abnormal activity leading to uncontrolled cell growth, cancer cell proliferation and survival. Kinase inhibitors target this dysfunction to disrupt cancer progression. In metastatic colorectal cancer (mCRC), regorafenib and fruquintinib are approved tyrosine kinase inhibitors used as third-line therapies when the disease has progressed. Regorafenib, an oral multi-kinase inhibitor, impedes angiogenesis, tumour growth, and the tumour microenvironment by inhibiting multiple signalling pathways, thus curtailing tumour advancement [45].

### Management of Metastatic CRC

Metastatic CRC is managed through a combination of chemotherapy and targeted therapies. Monoclonal antibodies that inhibit the epidermal growth factor receptor (EGFR), such as cetuximab and panitumumab, are used in patients with *KRAS* wild-type tumours, particularly in left-sided CRC. Additionally, bevacizumab, which targets vascular endothelial growth factor (VEGF), is employed to suppress angiogenesis and limit tumour progression. For cases amenable to surgical resection, especially in the liver, removing isolated metastases significantly improves outcomes, with a notable 5-year overall survival rate of approximately 20%. Surgical intervention for liver metastases broadens the therapeutic options available [8].

### Ongoing Clinical Trials on Management

Ongoing advancements in CRC management are supported by a variety of clinical trials exploring innovative therapeutic approaches (Table 1).

**Table 1:** Overview of vaccines for the management of colorectal cancer (CRC) currently in clinical trials. These vaccines employ various immunization strategies, such as DNA-based, peptide-based, dendritic cell, and neoantigen-based approaches, to enhance immune responses against CRC. The trials aim to assess safety, immunogenicity, efficacy, and potential for cancer treatment in different patient populations, including those with Lynch syndrome, advanced CRC, and high-risk adenomas

Study	Type of study	Aim	Trial identifier/ reference	Start date	Completion date
Assessment of Safety and Immunogenicity of Intradermal Electroporation of tetwtCEA DNA in Patients With Colorectal Cancer	Interventional, non-randomized, open label	To evaluate the safety and immunogenicity of a DNA immunization approach, where tetwtCEA DNA will be administered in combination with electroporation to CRC patients.	NCT01064375 [48]	2009	2016
Dendritic Cell Vaccination in Patients With Lynch Syndrome or Colorectal Cancer With MSI	Interventional, non-randomized, open label	To evaluate the safety and feasibility of vaccination with frameshift-derived neoantigen-loaded DC of CRC patients.	NCT01885702 [49]	2010	2024
Phase II Trial of Combination Immunotherapy in Subjects With Advanced Small Bowel and Colorectal Cancers	Interventional, non-randomized, open label	To investigate if a novel combination of vaccines can shrink tumours in people with advanced small bowel and CRC cancers.	NCT04491955 [50]	2020	2024
Vaccine Therapy in Treating Patients With Newly Diagnosed Advanced Colon Polyps	Interventional, randomized, double blind	To compare the immunogenicity a MUC1 peptide vaccine in participants with a history of an advanced adenoma, randomized to receive MUC1 peptide vaccine versus placebo.	NCT02134925 [51]	2014	2025
Cancer Preventive Vaccine Nous-209 for Lynch Syndrome Patients	Interventional, randomized, open label	To evaluate the safety, tolerability, and immune response of GAd-209-FSP and MVA-209-FSP vaccines in participants with Lynch syndrome.	NCT05078866 [52]	2022	2025
Testing a Combination of Vaccines for Cancer Prevention in Lynch Syndrome	Interventional, randomized, double blind	To evaluate if the combination of trivalent adenovirus-5 vaccines and IL-15 superagonist nogapendekin alfa inbakicept reduces the incidence of colorectal neoplasms in patients with Lynch syndrome.	NCT05419011 [53]	2023	2027
Neoantigen-based Peptide Vaccine and Conventional Third-line Therapy for CRC Progressed After Second-line Treatment	Interventional, non-randomized, open label	To investigate the response rate, control rate and safety of combined treatment of personalized tumour neoantigen-based peptide vaccine and conventional third-line therapy to patients with CRC progressed after second-line treatment	NCT06751966 [54]	2024	2027
Evaluation and exploration of the phase I/II clinical safety and efficacy of personalized FAST (Radiation fueled antigens stimulated T-cell immunotherapy) cancer vaccine combined with radiotherapy	Cohort, prospective	To evaluate the safety and surveillance using the patient’s individualized tumour tissue as tumour whole-cell vaccines vaccine and combine it with precision radiotherapy in patients who have filed third line of treatment.	NCT06756295 [55]	2025	2028
A Study of Encorafenib Plus Cetuximab With or Without Chemotherapy in People With Previously Untreated Metastatic Colorectal Cancer (BREAKWATER)	Interventional, randomized, open label	To evaluate the safety and efficacy of combining encorafenib and cetuximab, with or without chemotherapy, as a first-line treatment in patients with previously untreated BRAF V600E–mutant metastatic colorectal cancer.	NCT04607421 [56]	2020	2026

Numerous clinical trials are focusing on advancing CRC management through innovative vaccine-based immunotherapies targeting carcinoembryonic antigen (CEA) and other strategies [46, 47]. A notable trial completed in 2016 (NCT01064375), aimed to evaluate the safety and immune response of a CEA-targeting DNA vaccine in CRC patients, though its findings are yet to be published [48]. Similarly, a Phase I/II trial (NCT01885702) assessed dendritic cell vaccines loaded with neoantigens for CRC patients exhibiting microsatellite instability or possessing specific genetic mutations, with results still pending [49].

In 2024 a Phase II trial (NCT04491955) explored of the potential of combining immunotherapy agents, including CV301 and various cancer vaccines, to effectively reduce advanced tumour burden [50]. Meanwhile, an ongoing Phase II study (NCT02134925) investigates an MUC1 peptide vaccine designed to prevent the recurrence of adenomatous polyps in CRC patients [51].

Focusing on Lynch syndrome, a Phase Ib/II trial (NCT05078866) is examining the Nous-209 vaccine’s ability to prevent cancer progression by training the immune system to target neoantigens, with the study expected to conclude in 2025 [52]. Additionally, a Phase IIb trial (NCT05419011) is testing a combination of the Tri-Ad5 vaccine and N-803 to strengthen immune responses in Lynch syndrome, with a projected completion date in 2027 [53].

Concurrently, other trials (NCT06751966, NCT06756295) are evaluating personalized neoantigen-based vaccines and Radiation-Fueled Antigens Stimulated T-cell Immunotherapy for treatment-resistant tumors, aiming to extend survival outcomes [54, 55]. These research efforts collectively represent a significant push towards developing targeted immunotherapies for various CRC patient populations.

The BREAKWATER trial (NCT04607421) is a phase III study evaluating the combination of encorafenib (a BRAF inhibitor) and cetuximab (an anti-EGFR antibody), with or without chemotherapy, in patients with BRAF V600E-mutant metastatic colorectal cancer (mCRC). The trial aims to assess progression-free survival (PFS) and overall survival (OS), seeking to establish a new standard of care for this poor-prognosis patient subgroup [56].

### Prevention

#### Primary Prevention

Primary prevention refers to actions or measures taken to prevent the development of a disease or health condition before it manifests. Primary prevention actions in CRC are mostly focused on promoting healthy dietary habits, encouraging physical activity, and cessation of smoking [57].

#### Healthy Diet

Dietary habits significantly influence the development of CRC. Excessive caloric intake, often linked to obesity, increases CRC risk, while calorie restriction may help reduce this risk by mitigating obesity-related factors [58]. Furthermore, a clear link has been found between the consumption of red and processed meats and a higher CRC risk. A UK-based study revealed that individuals consuming 76 g/day of red and processed meat had a 20% higher CRC risk than those consuming 21 g/day [59]. Harmful substances including heterocyclic amines, formed during high-temperature meat cooking, can also increase CRC risk, particularly in genetically predisposed individuals [60]. Conversely, higher fiber intake, especially from bread and cereals, reduces CRC risk, with a 14% lower risk found in individuals consuming more fiber [59, 61]. However, the protective effect of fiber may be reduced with other risk factors such as high red meat or alcohol intake, which has been linked to an 8% increased CRC risk for every 10 g/day consumed [59].

#### Preliminary Results on the Association between Nutritional Supplements and CRC Risk

Research has explored the potential role of medications and supplements in the primary prevention of CRC, though these findings are preliminary and not part of formal guidelines.

#### Calcium

Epidemiological and mechanistic evidence suggests that calcium may reduce CRC risk, with meta-analyses showing a 9% reduction in risk for every 300 mg/day increase in calcium intake (RR = 0.91, 95% CI: 0.86–0.98) [62]. A retrospective study found that higher calcium intake (≥ 1400 mg/day vs. <600 mg/day) was associated with a lower risk of colon cancer (RR = 0.78, 95% CI: 0.65–0.95). The protective effect was strongest when calcium intake occurred 12–16 years before diagnosis (RR = 0.76, 95% CI: 0.64–0.91). Despite these promising findings, inconsistencies in trial designs prevent universal recommendations. Further research is needed to clarify calcium’s role in CRC prevention and establish supplementation guidelines [63].

#### Vitamin D

High doses of vitamin D have been associated with a decreased risk of CRC in some studies, while lower levels may not be effective [59]. Despite these associations, more research is needed to establish definitive guidelines for vitamin D supplementation as a preventive measure.

#### Physical Activity

Regular physical activity is estimated to reduce the risk of CRC by about 40%, regardless of BMI. Such activity improves weight management, lowers insulin and inflammation levels, boosts immune function, and improves digestive health, all of which help prevent cancer. Obesity, on the other hand, raises cancer risk primarily through negative metabolic effects such as elevated insulin and chronic inflammation, both of which have been shown to promote cell growth by creating an ideal microenvironment for tumors to thrive [59, 64].

### Preliminary Results on the Association between Drugs and CRC Risk

#### Aspirin and NSAID

Regular use of aspirin and nonsteroidal anti-inflammatory drugs (NSAIDs) has been linked to a lower risk of CRC. A cohort study of over 2.1 million individuals followed for a median of 10.9 years found that current low-dose aspirin use reduced CRC risk by 13% (HR 0.87; 95% CI 0.84–0.90) compared to non-use. The reduction was most significant for metastatic CRC (HR 0.79) and increased with longer use, with a 16% lower risk after ≥ 5 years (P value < 0.001). Aspirin use during the study period was estimated to have averted 1,073 CRC cases (95% CI 818–1,338), highlighting its preventive potential [65]. However, the potential risks of long-term aspirin use, such as gastrointestinal bleeding, must be carefully weighed against the benefits.

#### Hormonal Therapy

There is evidence of an inverse relationship between hormonal therapy and the risk of CRC in postmenopausal women. This preventive benefit appears to diminish three years after discontinuation of the treatment [66].

### Secondary Prevention

Secondary prevention involves interventions aimed at detecting and treating a disease or health condition in its early stages to prevent its progression and reduce complications [57].

#### Stool Testing and Endoscopic Evaluations

Stool testing and endoscopic large bowel evaluations are standard methods for CRC screening. Alternatives such as capsule endoscopy, CT scans, stool-based tests, blood tests, are less competitive due to lower diagnostic accuracy, cost-effectiveness, or adverse effects associated with CT. However, global efforts are underway to identify novel biomarkers to enhance minimally invasive CRC screening options [67].

Newer fecal immunochemical tests (FIT) offer benefits over older guaiac fecal occult blood tests (gFOBT), such as greater specificity for human hemoglobin and no dietary restrictions. FITs require only one stool sample and have higher adherence rates. They also show significantly greater sensitivity for detecting CRC (60–80% for FITs vs. 30–40% for gFOBTs) and advanced adenomas (20–30% for FITs vs. 10% for gFOBTs) [68–70].

#### Annual Screening

Screening for CRC is recommended for everyone who is aged 45 to 75 and is at average risk. Individuals at higher risk, such as those with a family history of CRC, should start screening earlier, around the age of 40. Several screening methods are recommended, including a colonoscopy every 10 years for average-risk individuals and every 5–10 years for high-risk individuals; annual FIT screening for those at average risk and every 1–2 years for high-risk groups; and CT every five years as an alternative [71]. Annual screening for CRC combined with colonoscopy follow-up for positive findings and lesion excision, has been shown to reduce CRC incidence and mortality by 20–30% [67].

#### Disparities in Access To Screening Programs

Despite the worldwide burden of CRC, screening rates are low in low- and middle-income countries and lower-income minorities within advanced economies. Observational studies have shown that these groups have higher CRC incidence, but low income, low education, poor awareness, and lack of social security are barriers that limit access to and use of screening examinations [72]. Currently, there are multiple effective CRC screening methods including colonoscopy, sigmoidoscopy, fecal occult blood tests (FOBT), FIT, and Computed tomography (CT) colonography, but many low-income countries still lack established CRC screening programs and consistent screening guidelines [72].

#### Ongoing Trials on Screening

Several ongoing clinical trials are exploring innovative methods to improve CRC screening, emphasizing accuracy, adherence, and accessibility. These efforts aim to refine screening processes by focusing on population-specific needs and enhancing detection methodologies (Table 2).

**Table 2:** Overview of clinical trials investigating colorectal cancer (CRC) screening methods as secondary prevention. These trials assess various screening techniques, including colon capsule endoscopy, optical colonoscopy, fecal immunochemical testing (FIT), and blood-based multiomics testing. The studies aim to evaluate the effectiveness, adherence, and accuracy of these screening approaches in different populations, including average-risk individuals, first-degree relatives of CRC patients, and underserved communities

Study	Type of study	Aim	Trial identifier/ reference	Start date	Completion date
The Efficiency of Colon Capsule Endoscopy in Colon Cancer Screening	Interventional, open label	To enhance colorectal cancer screening in the Czech Republic by reducing incidence and mortality, and to assess the effectiveness of a new minimally invasive device in prevention.	NCT03052335 [73]	2017	2019
Comparison Colon Capsule Endoscopy vs. Optical Colonoscopy for Colorectal Cancer Screening in Familiar-Risk Population	Interventional, randomized, open label	To compare adherence and effectiveness of colon capsule endoscopy versus conventional colonoscopy for screening first-degree relatives of colorectal cancer patients.	NCT01557101 [74]	2012	2013
Prevention of Colorectal Cancer Through Multiomics Blood Testing	Cohort, prospective	To validate the accuracy of a blood-based test for early colorectal cancer detection in average-risk individuals undergoing routine screening colonoscopy. participants undergoing routine screening colonoscopy	NCT04369053 [75]	2020	2024
Colorectal Cancer Screening in Average-risk Population: a Multicenter, Randomized Control Trial Comparing Immunochemical Fecal Occult Blood Testing Versus Colonoscopy	Interventional, randomized, open label	To compare the efficacy of biennial iFOBT versus colonoscopy every 10 years in reducing colorectal cancer mortality and to assess compliance and complications in an average-risk population	NCT00906997 [76]	2008	2021
A trial comparing the effectiveness of two strategies for distributing fecal immunochemical test kits in a community-based colorectal cancer screening program targeting African Americans	Interventional, non-randmized, single participant	to determine whether combining on-site FIT kit distribution with social media advertising increases return rates compared to on-site distribution alone and to identify the more effective strategy for improving colorectal cancer screening among underserved populations	NCT05903885 [77]	2023	2026

One study (NCT03052335) evaluates second-generation colon capsule endoscopy (CCE2) as a non-invasive option for those with positive fecal immunochemical test (FIT) results, aiming to decrease reliance on traditional colonoscopies by assessing its effectiveness and cost-efficiency compared to standard methods [72].

Another trial (NCT01557101) assessed increased adherence to CRC screening among first-degree relatives of CRC patients using CCE2, looking to improve participation rates in high-risk groups, though its results remain unpublished [74].

The PREEMPT CRC study (NCT04369053) completed in 2024, validated a blood-based test using machine learning to analyze cell-free biomarkers for early CRC detection, with outcomes pending [75]. Furthermore, a Spanish randomized controlled trial compared biennial FIT and decennial colonoscopy to evaluate impacts on CRC mortality, concluding in 2021 with results forthcoming (NCT00906997) [76]. Additionally, a non-randomized trial (NCT05903885) targets screening among African Americans by comparing direct FIT kit distribution with social media advertising, aiming to enhance participation and decrease racial disparities in screening, expected to conclude in 2026 [77].

#### Tertiary Prevention

Tertiary prevention involves strategies and interventions aimed at minimizing the impact of an existing disease, preventing complications, and improving the quality of life for individuals already diagnosed with the condition. The factors associated with CRC risk also have implications for patient survival, providing opportunities for tertiary prevention [57].

#### Healthy Nutrition

Adhering to a healthy lifestyle post-diagnosis, which involves the consumption of a healthy diet consistent with WCRF guidelines, is associated with longer survival in stage-III colon cancer patients [78, 79].

#### Physical Activity

Physical activity has a beneficial effect on CRC outcomes, reducing symptoms, improving quality of life, and increasing survival. The postulated mechanisms include fat loss, inflammation control, metabolic modulation, and better immunity [80].

#### Smoking and Alcohol Intake

Smoking and excessive alcohol intake correlate with diminished survival rates, likely attributed to increased surgical complications, decreased efficacy of therapy, and the impact of nicotine on cancer cells [81].

#### Aspirin

Aspirin has shown potential in CRC prevention and progression via COX and non-COX pathways. Low-dose aspirin may reduce CRC risk and improve survival and normalize tissue markers. It also decreases prostaglandin E2 levels, improving treatment response [82, 83]. Post-diagnosis, aspirin appears to enhance survival, particularly in cases with COX-2 expression and PIK3CA mutations, and can reduce circulating tumour cells and adenoma recurrence [84]. Despite these promising results, aspirin’s role in CRC prevention and treatment is not yet standardized in guidelines, and further research is needed to confirm its clinical utility.

#### Vitamin D

A recent phase II clinical trial (SUNSHINE) reported that a high-dose vitamin D supplementation preliminarily increased progression-free survival (PFS) in metastatic CRC, [85]. On the contrary, the larger SOLARIS phase III study failed to demonstrate a statistically significant improvement in PFS, indicating no overall benefit in unselected patients [86].

## Conclusions

Addressing the growing burden of CRC requires a multifaceted approach integrating prevention, early detection, and innovative treatment strategies. The rising global incidence, largely driven by lifestyle factors and aging populations, underscores the need for targeted public health initiatives. Encouraging dietary improvements, physical activity, and widespread awareness campaigns can significantly reduce risk factors and promote early diagnosis through routine screenings.

Enhancing screening accessibility, particularly for underserved populations, is critical. Leveraging advanced technologies, such as AI-assisted imaging, can improve detection accuracy and streamline diagnostic processes. Additionally, ongoing research into novel biomarkers and personalized treatment strategies, including cancer vaccines and targeted therapies, holds promise for refining CRC management.

Future advancements in CRC treatment will focus on expanding precision medicine approaches, such as anti-PD-1 immunotherapy for localized dMMR tumors, *KRAS* inhibitors for specific mutations, and novel combinations of immunotherapy for MSS CRC. Additionally, targeted therapies including *BRAF* inhibitors, currently under investigation in trials such as BREAKWATER, hold promise for improving outcomes *in BRAF-mutant* CRC. By translating these advancements into clinical practice and integrating precision medicine with public health initiatives, significant progress can be made in reducing the burden of CRC through more effective prevention, detection, and treatment strategies.

## Data Availability

No datasets were generated or analysed during the current study.

## Key References

- Siegel RL, Wagle NS, Cercek A, Smith RA, Jemal A. Colorectal cancer statistics, 2023. CA Cancer J Clin. 2023;73(3):233–54. doi: 10.3322/caac.21772.
  - This paper provides statistical data on colorectal cancer in 2023. Understanding these statistics is crucial for identifying trends in incidence and mortality, which helps in guiding preventive measures and early detection strategies globally.
- Pleguezuelos-Manzano C, Puschhof J, Rosendahl Huber A, van Hoeck A, Wood HM, Nomburg J, Gurjao C, et al. Mutational signature in colorectal cancer caused by genotoxic *pks*⁺ *E. coli*. *Nature*. 2020;580(7802):269. doi: 10.1038/s41586-020-2080-8.
  - This study introduces a new mutational signature caused by the genotoxic *pks⁺ E. coli*, providing insight into one of the potential microbiome influences on colorectal cancer. This could lead to novel preventive strategies targeting the microbiome.
- Argiles G, Tabernero J, Labianca R, Hochhauser D, Salazar R, Iveson T, et al. Localised colon cancer: ESMO clinical practice guidelines for diagnosis, treatment and follow-up. Ann Oncol. 2020;31(10):1291–1305. doi: 10.1016/j.annonc.2020.06.022.
  - This paper presents the European Society for Medical Oncology (ESMO) guidelines for the management of localized colon cancer, providing up-to-date recommendations on diagnosis, treatment, and follow-up care. These guidelines are pivotal for oncologists in providing evidence-based care.
- Duan B, Zhao Y, Bai J, Wang J, Duan X, Luo X, Zhang R, et al. Colorectal cancer: an overview. Exon Publications; 2022:1–2.
  - This comprehensive overview provides a broad understanding of colorectal cancer, including its pathophysiology, risk factors, and management. It serves as a useful resource for both clinicians and researchers looking for a general overview of the disease.
- Holtedahl K, Borgquist L, Donker GA, Buntinx F, Weller D, Campbell C, et al. Symptoms and signs of colorectal cancer, with differences between proximal and distal colon cancer: a prospective cohort study of diagnostic accuracy in primary care. BMC Fam Pract. 2021;22:1–3.
  - This study focuses on the diagnostic accuracy of symptoms in primary care, emphasizing the differences in presentation between proximal and distal colon cancers. It highlights the importance of early recognition in primary care settings to improve outcomes through early referral.
- Syed AR, Thakkar P, Horne ZD, Abdul-Baki H, Kochhar G, Farah K, Thakkar S. Old vs. new: Risk factors predicting early onset colorectal cancer. World J Gastrointest Oncol. 2019;11(11):1011.
  - This paper discusses the changing landscape of colorectal cancer risk factors, particularly focusing on the rise of early-onset colorectal cancer. Understanding these new risk factors is essential for identifying high-risk populations and guiding screening initiatives.
- Sung H, Ferlay J, Siegel RL, Laversanne M, Soerjomataram I, Jemal A, et al. Global cancer statistics 2020: GLOBOCAN estimates of incidence and mortality worldwide for 36 cancers in 185 countries. CA Cancer J Clin. 2021;71(3):209 − 49. doi: 10.3322/caac.21660.
  - This paper provides global cancer statistics, including data on colorectal cancer, which is critical for understanding global trends in incidence, mortality, and the need for targeted interventions, particularly in low- and middle-income countries.
- Morgan E, Arnold M, Gini A, Lorenzoni V, Cabasag CJ, Laversanne M, Vignat J, et al. Global burden of colorectal cancer in 2020 and 2040: incidence and mortality estimates from GLOBOCAN. Gut. 2023 Feb 1;72(2):338 − 44.
  - This paper projects the global burden of colorectal cancer up to 2040, providing valuable data on future incidence and mortality trends. This information is key for public health planning and resource allocation to address the rising burden of colorectal cancer globally.
- Morgan E, Arnold M, Gini A, Lorenzoni V, Cabasag CJ, Laversanne M, Vignat J, et al. Global burden of colorectal cancer in 2020 and 2040: incidence and mortality estimates from GLOBOCAN. Gut. 2023 Feb 1;72(2):338 − 44.
  - This paper projects the global burden of colorectal cancer up to 2040, providing valuable data on future incidence and mortality trends. This information is key for public health planning and resource allocation to address the rising burden of colorectal cancer globally.
- Fan A, Wang B, Wang X, Nie Y, Fan D, Zhao X, et al. Immunotherapy in colorectal cancer: current achievements and future perspective. Int J Biol Sci. 2021;17(14):3837.
  - This paper discusses recent advancements in immunotherapy for colorectal cancer, an area of great promise for treatment. It provides a review of the current status of immunotherapy and highlights future research directions, making it highly relevant to ongoing clinical trials and treatment strategies.
